# Knee pain and health-related quality of life among older patients with different knee osteoarthritis severity in Saudi Arabia

**DOI:** 10.1371/journal.pone.0196150

**Published:** 2018-05-15

**Authors:** Saad M. Bindawas, Vishal Vennu, Saud Alfhadel, Ali D. Al-Otaibi, Ahmad S. Binnasser

**Affiliations:** 1 Department of Rehabilitation Sciences, College of Applied Medical Sciences, King Saud University, Riyadh, Saudi Arabia; 2 Physical Therapy Department, General Directorate of Medical Services, Riyadh, Saudi Arabia; 3 Physical Therapy Department, Dawadmi General Hospital, Riyadh, Saudi Arabia; 4 Department of Orthopedic, College of Medicine, King Saud University, Riyadh, Saudi Arabia; Xuanwu Hospital, Capital Medical Universty, CHINA

## Abstract

**Objective:**

There is a lack of knowledge about health-related quality of life (HRQoL) in Saudi patients with musculoskeletal impairment, particularly among older adult populations. Thus, the current research aimed to determine the association of knee osteoarthritis (OA) severity with knee pain (KP) and HRQoL among older patients in Riyadh, Saudi Arabia.

**Methods:**

In a multicenter cross-sectional study, we recruited 209 consecutive males and females aged ≥55 years with radiographically diagnosed knee OA from five hospitals across Riyadh, Saudi Arabia. According to the Kellgren & Lawrence classification, patients were classified into two groups: mild/moderate knee OA (n = 126) and severe knee OA (n = 83). KP and HRQoL were assessed using the pain visual analogue scale (VAS) and the 36-Item Short Form Health Survey (SF-36), respectively. A higher score on the pain VAS and the SF-36 represented worse KP and better HRQoL, respectively.

**Results:**

Severe knee OA was significantly associated with an increased score of 3.47 (*p* <.0001) points on the pain VAS compared with the score reported by patients with mild/moderate knee OA. Additionally, it was significantly associated with reduced scores of 6.83 and 5.82 (both: *p* <.0001) points on the physical and mental composite summary subscales of the SF-36, respectively, compared with the scores of patients with mild/moderate knee OA, even after adjusting for all covariates.

**Conclusion:**

Older patients with severe knee OA had significantly worse KP and reduced HRQoL compared to patients with mild/moderate knee conditions, even after controlling for confounders.

## Introduction

Knee osteoarthritis (OA) is a degenerative disease affecting not only the articular cartilage but also the entire joint [1]. It has been listed as the 11^th^ largest contributor to global disability and the 38^th^ highest contributor in terms of disability-adjusted life years [2]. Approximately 250 million (3.6% of the population) people worldwide have had knee OA [2]. The majority of them living in low- and middle-income countries with a moderate to severe knee OA compared to those with mild knee OA [2]. Most of the data on knee OA severity are available from few high-income countries [3].

Saudi Arabia is the most prominent country (2,150,000 square kilometers) in the Arabian Peninsula with a population of more than 32 million [4]. People of this county culture differ from Western culture in their daily activities, such as praying, ablution and sitting to eat on the floor. Most of these events require full knee flexion, and knee flexibility significantly decreased in patients with knee OA [5]. It has been reported that knee OA is a rapidly growing health problem in people over 50 years of age and a significant cause of disability [6, 7]. Therefore, there is a need to assess the health status and function of these patients, taking into account the activities specific to Saudi culture.

Previously published studies have shown that the presence of pain in patients with knee OA led to a decrease in health-related quality of life (HRQoL) [8–12]. Consistent with these studies, measuring HRQoL is essential for patients with severe knee OA in Saudi Arabia. It is not only central to describing the impact of a disease or intervention, but it also aids in informed decision-making regarding an allocation of often limited healthcare resources [13]. It is particularly appropriate to evaluate the patient's current levels of functioning and perceived well-being regarding physical and mental health [14]. In various sociocultural contexts, a qualitative systematic literature review was performed to identify relevant domains/items of HRQoL for patients with knee OA [15]. Surprisingly, it has been reported that the essential HRQoL domains/items for those patients are limited in the United States, Canada, Ireland, France, Sweden, and the United Kingdom.

To the best of our knowledge, the relationships between knee OA severity, knee pain (KP), and HRQoL have not been thoroughly studied in Saudi Arabia. Thus, the current research aimed to examine the associations of knee OA severity with KP, and HRQoL among patients in Saudi Arabia. We hypothesized that severe knee OA would be associated with worse KP and reduced HRQoL.

## Materials and methods

### Study design and setting

A cross-sectional study was conducted between March 2016 and March 2017in the orthopedic and physiotherapy departments of the following five hospitals: King Saud University Medical City (KSUMC), King Faisal Specialist Hospital & Research Center (KFSHRC), King Saud Medical City (KSMC), Dwadmi General Hospital (MOHDH) and Quwaieah General Hospital (MOHQH), Riyadh, Saudi Arabia.The study was carried out as per the Declaration of Helsinki, and the study protocol was approved by the Research Ethics Committee and the Institutional Review Boards (IRBs) of the College of Applied Medical Sciences, KSUMC, KFHSRC, KSMC, MOHDH, and MOHQH. A written informed consent was collected from each participant upon enrollment in the study.

### Patients and inclusion and exclusion criteria

All the patients (n = 209), males and females aged 55 years and above with radiographically diagnosed knee OA confirmed by physicians according to the standards of the American College of Rheumatology, were included in the current study. Patients’ knee OA severity was defined according to the Kellgren-Lawrence (KL) scale proposed by Kellgren and colleagues [16]. According to the proposed KL classification, patients with knee OA were divided into two groups: mild/moderate knee OA (n = 126) and severe knee OA (n = 83). Patients with severe rheumatoid arthritis or fractures and those who had received major surgery on lower limbs or an intra-articular injection in the last six months were excluded.

### Outcome measures

KP was assessed in the most recent 24 hours using the pain visual analog scale (VAS), a single-item continuous scale composed of a horizontal or vertical line. The score is determined by measuring the distance (in mm) on a 10-cm line between the "no pain" (score of 0) anchor and the patient’s mark (evaluating their pain), providing a range of scores from 0–10. A higher score indicates higher pain intensity. The reliability and construct validity of this scale were sufficient to measure the pain of adults [17].

HRQoL was evaluated using the reliable and valid Saudi Arabia version of 36-Item Short Form Health Survey (SF-36), a generic, self-administered, and patient-reported outcome measure [18]. It consists of 36 items in 8 domains that assess the patient's physical and mental status. The Physical Composite Scale (PCS) includes the 4 physical domains, such as physical functioning (PF) (10 items), role limitations due to physical functioning (PF) (4 items), bodily pain (BP) (2 items), and general health (GH) (5 items). The Mental Composite Scale (MCS) include the 4 mental domains, such as vitality (VT) (4 items), social functioning (SF) (2 items), role limitation due to emotional problems (RE) (3 items), and mental health (MH) (5 items). The PCS and MCS scores vary from 0 to 100, with higher ratings indicating a better HRQoL.

### Variables

All information regarding each patient’s social-demographic characteristics such as age, gender (male and female), education (primary school or less and high school or more), and employment status (employed and self-employed/retired) were collected using a specific form designed for this research. Clinical test results, such as knee OA severity, affected knee with OA, the duration of knee OA (in years), and body mass index (BMI), calculated as the weight in kilograms (kg) divided by height squared in square meters (m^2^), were recorded for each patient.

### Statistical analysis

Descriptive statistics, stratified by the severity of knee OA, are presented as the counts (percentages), means and standard deviation (SD). Chi-square tests for categorical variables and two independent sample t-tests for continuous variables were used to examine the associations according to the severity of knee OA. Linear regression was used to investigate the relationship between knee OA severity, KP, and HRQoL.

The relationship was investigated with unadjusted and adjusted analyses. In unadjusted analyses, the association was examined with no other covariates. In adjusted analyses, the association was tested in the presence of covariates, such as age, gender, education, employment status, BMI, knee involvement and duration of knee OA. Mild/moderate knee OA was used as a reference. A full anonymised data set is provided as a supplementary file (S1 Dataset). All the analyses were performed using the statistical analysis software (SAS) for Windows, version 9.2 (SAS Institute, Inc., Cary, NC, USA).

## Results

The characteristics of the total sample population are exhibited in Table 1. The average age of the whole sample was 59.4 years (SD = 7.4). Patients with severe knee OA were, on average, one year older than those with mild/moderate knee OA. The majority of the patients with severe knee OA were female (73%), had primary school or less education (51%), and were self-employed or retired (76%). Most patients with severe knee OA had knee OA in both knees (83%), high body mass index (BMI) scores (33.5 ± 5.7), long duration of knee OA (average 7.5 years), and high pain VAS scores (8.2 ± 0.9).

**Table 1:** The total sample population characteristics (n = 209).

Characteristics	Mild/Moderate knee OA N = 126 (60%)	Severe knee OA N = 83 (40%)	*P-Value*
**Age in years**	58.9 ± 7.9	60.0 ± 7.0	.391
**Gender**			.022
Male	53 (42)	22 (27)	
Female	73 (58)	61 (73)	
**Education**			.002
Primary school or less	32 (25)	42 (51)	
High school or more	94 (75)	41 (49)	
**Occupation**			.554
Employed	35 (28)	20 (24)	
Self-employed or retired	91 (72)	63 (76)	
**Affected Knee with OA**			.498
Right/left	26 (21)	14 (17)	
Both	100 (79)	69 (83)	
**Body Mass Index (kg/m**^**2**^**)**	31.9 ± 5.3	33.5 ± 5.7	.040
**Duration of knee OA in years**	4.2 ± 4.5	7.5 ± 4.9	<.0001
**VAS for pain**	4.5 ± 1.2	8.2 ± 0.9	<.0001

OA = osteoarthritis, VAS = visual analogue scale. Values are present as the mean ± standard deviation or count (percentage).

**Fig 1:**
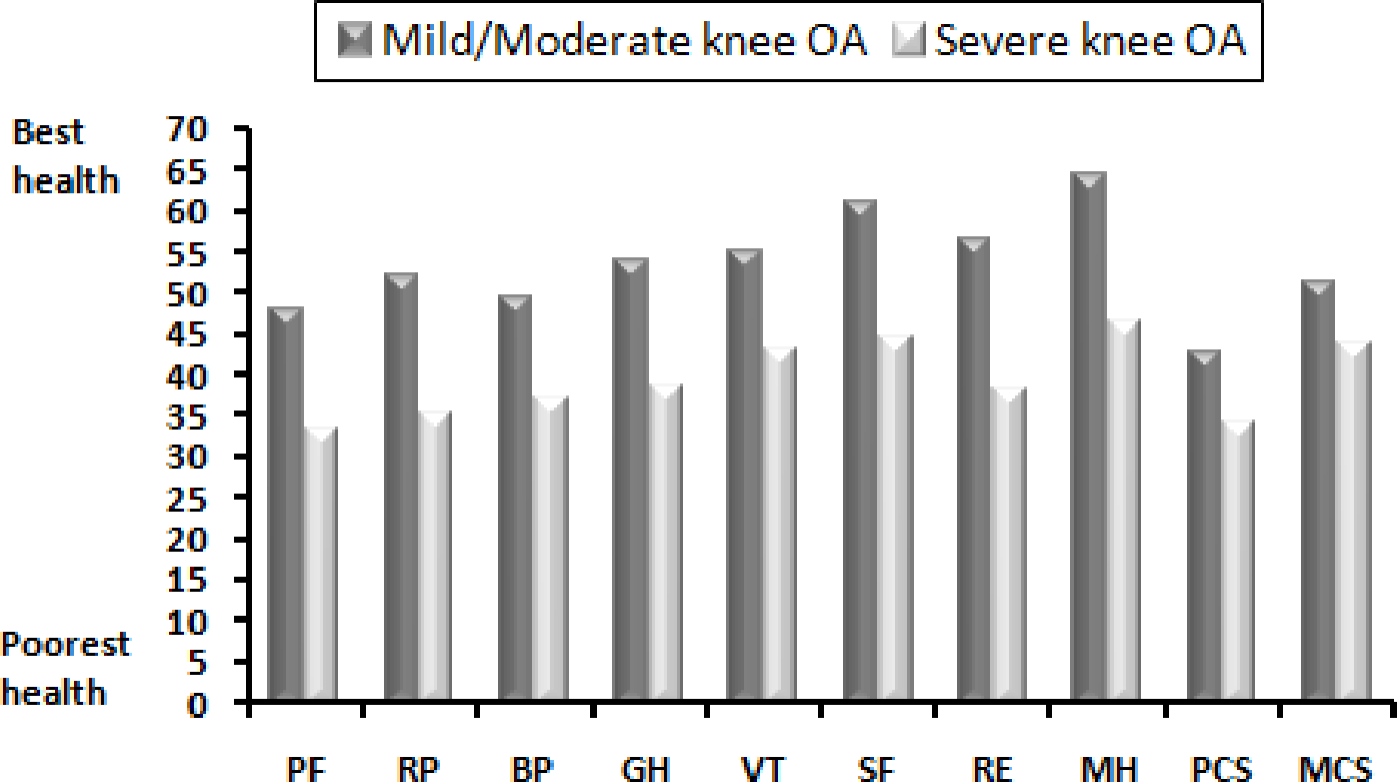
The mean SF-36 subscale scores comparison with knee osteoarthritis severity. Two independent sample t-test, *P<*.*0001*. PF = physical functioning, RP = role limitations due to physical functioning, BP = bodily pain, GH = general health, VT = vitality, SF = social functioning, RE = role limitations due to emotional problems, MH = mental health, PCS = physical component summary, MCS = mental component summary.

Fig 1 shows the mean domain and composite SF-36scores for patients according to knee OA severity. Patients with severe knee OA reported significantly lower scores in all eight domains of the SF-36 than those with mild/moderate knee OA. Most domain scores were smaller than the standard normative value of 50 (SD = 10), indicating reduced HRQoL among OA patients in Saudi Arabia.

Regression analyses of the association between severe knee OA, KP, and HRQoL are shown in Table 2. Patients with severe knee OA were significantly associated with an increased score of 3.68 (*p* <.0001) points on the pain VAS relative to the score of those with mild/moderate knee OA. The association remained significant (β = 3.47 points, *p* <.0001) after adjusting for all variables such as age, gender, education, employment status, involved knee with OA, BMI, and duration of knee OA.

**Table 2:** Regression analysis of the association between severe knee osteoarthritis, knee pain, and health-related quality of life.

Dependent variables		n	β	SE	95% CI	*P-Value*
Pain VAS	Unadjusted	209	3.68	0.16	3.35 to 4.00	<.0001
	Adjusted	206	3.47	0.17	3.12 to 3.82	<.0001
PCS	Unadjusted	209	-8.43	0.92	-10.27 to -6.60	<.0001
	Adjusted	206	-6.83	0.94	-8.70 to -4.96	<.0001
MCS	Unadjusted	209	-7.39	1.29	-9.93 to -4.86	<.0001
	Adjusted	206	-5.82	1.37	-8.53 to -3.12	<.0001

n = number of patients; SE = standard error; VAS = visual analogue scale; CI = confidence interval; PCS = physical composite summary; MCS = mental composite summary.

Reference: mild/moderate knee osteoarthritis.

Unadjusted = included severity of knee osteoarthritis plus age, gender, education, and employment status.

Adjusted = included severity of knee osteoarthritis plus age, gender, education, employment status, involved knee with osteoarthritis, BMI, and duration of knee osteoarthritis.

Patients with severe knee OA showed significantly reduced SF-36 subscale scores, with decreases of 8.43 points on the PCS and 7.39 points on the MCS (both: *p<*.0001), compared to the scores of patients with mild/moderate knee OA. After adjustment for both sociodemographic and clinical variables, these patients again showed significantly reduced scores, with decreases of 6.83 and 5.82 points on the PCS and MCS subscales of the SF-36, respectively (both: *p* <.0001), compared to the scores of patients with mild/moderate knee OA.

## Discussion

The current study examined the associations of knee OA severity with KP and HRQoL among patients in Saudi Arabia. Our findings demonstrated that patients with severe knee OA significantly showed an increased score on the pain VAS and reduced scores on the PCS and MCS subscales of the SF-36 compared to patients with mild/moderate knee OA even after adjusting for sociodemographic and clinical variables.

Furthermore, the current study also revealed that patients with severe knee OA were 1 year older on average than those with mild/moderate knee OA. In addition, they had knee OA for an average of 7.5 years and reported more KP than those with mild/moderate knee OA for 4 years with slight knee pain. Similar to our study variables, other recent studies [10, 19] and one epidemiological study [20] also found relationships between radiographic knee OA, age, BMI, duration of knee OA, and pain severity.

In this study, the results regarding the KP patterns and HRQoL are consistent with those of qualitative and quantitative studies [21–23]. According to these studies, radiographic progression of knee OA is correlated with a shift from intermittent pain alone to severe pain. Another study from Japan examined how the KP components varied depending on the severity of the radiographic knee OA [24]. A previously published study demonstrated that poorest HRQoL in patients with knee OA was also associated with having pain in both knees compared to patients with unilateral or no KP[9]. A population-based study in Japan revealed that patients with severe knee OA had a significantly lower physical quality of life than those with mild and moderate knee OA [25]. A large population-based cohort study from southern Sweden confirmed that participants with knee OA defined either clinically or radiographically reported lower HRQoL scores than those with no knee OA [8]. The results of another study showed that patients with radiographic knee OA had considerably lower scores in all subgroups of SF-36 compared with healthy controls[26]. The results obtained from a cross-sectional study revealed that the worst score on the PCS of the SF-36 subscale was inversely associated with increased pain severity in patients with knee OA [27].

However, those studies varied in methodology and were different from this research. This study evaluated the associations between knee OA severity, KP, and HRQoL among patients in Saudi Arabia. The findings of this study showed that KP severity in patients with knee OA varied with radiographic disease severity. Therefore, it suggested that worse KP in severe knee OA may indicate that it is more crucial to seek medical care to improve HRQoL compared to less KP in mild/moderate knee OA.

Our study has some limitations similar to any other study [28]. The data were examined cross-sectional, the association between knee OA severity, KP, and HRQoL cannot be viewed as causal. Longitudinal studies are needed to examine the relationship between knee OA severity, KP, and HRQoL. Another weakness of our study was that it relied on self-evaluated KP and self-reported HRQoL. Some patients might have had KP but did not have their information reported at the time of the interview, including their HRQoL; therefore, they were misclassified. Our investigation has several strengths, including that it is a multi-center study. We used a widely accepted and validated measure to assess KP, utilizing the pain VAS and HRQoL determined with the SF-36. We also used criteria provided by the American College of Rheumatology to define knee OA.

Previous studies have reported that knee OA is one of the most prevalent and growing health conditions in Saudi Arabia [7]. However, the health status and function of patients with knee OA in western countries were assessed using their measures that were explicitly developed for their cultures[29, 30]. These measures do not take into account activities specific to Saudi cultures, such as praying, ablution and sitting and eating on the floor [30, 31]. Moreover, some of these measures are limited in covering vast aspects of activities as recommended by the World Health Organization International Classification of Functioning, Disability, and Health (ICF) [30]. Therefore, a better understanding of the relationship between KP and HRQoL is necessary for policymakers, researchers, and clinicians to manage KP prevent knee OA and improve each patient’s HRQoL [32]. The current study findings provide knowledge regarding the effects of these conditions on HRQoL, and these findings are essential in the care and education of people with knee OA, which would further lead to excellent pain control and correct disability [7]. Furthermore, this study emphasizes the need for a new Arabic patient-reported outcome measure using the ICF framework to measure functions, disability, and health among Saudi patients.

In conclusion, older patients with severe knee OA had significantly worse KP and reduced HRQoL compared to patients with mild/moderate knee conditions, even after adjusting for sociodemographic and clinical variables. Other interesting findings include the fact that all patients were obese, and patients with severe knee OA reported more KP than those with mild/moderate knee OA did. Future studies at the national level are necessary to measure functioning, disability, and health for this patient population.

## Supporting information

S1 Dataset The full dataset has been provided on which all calculations have been based. (XLSX)
